# Assessing the Impact of a Red Trauma Simulation on Anesthesia Residents’ Confidence, Comfort, and Overnight Call Readiness

**DOI:** 10.7759/cureus.93102

**Published:** 2025-09-24

**Authors:** Robb Wasserman, Bryan Obika, Alexander S Doyal, Elisa Takalo, Vishal Dhandha

**Affiliations:** 1 Anesthesiology, University of North Carolina School of Medicine, Chapel Hill, USA

**Keywords:** anesthesia residency, massive blood transfusion, massive transfusion protocol, medical resident education, perceived confidence, trauma simulation

## Abstract

Introduction

The transition from intern year to the first year of anesthesia residency is a critical period marked by a steep learning curve and the need to develop clinical proficiency rapidly. Managing overnight call, where residents face high-stakes, emergent situations, is particularly challenging. Confidence and comfort during these calls are essential for both patient safety and resident well-being. The primary objective is to evaluate whether participation in this simulation improves confidence and comfort levels of new anesthesia residents.

Methods

Thirteen first-year clinical anesthesia (CA-1) residents participated in the simulation of a 25-year-old male trauma patient after an all-terrain vehicle (ATV) accident with a positive focused assessment with sonography for trauma (FAST) exam and deteriorating vital signs. The simulation progressed through stages of induction, worsening hemodynamic instability, and resuscitation using a massive transfusion protocol (MTP). A simulation mannequin and monitors were used to accurately portray a realistic operating room. Baseline data on residents’ comfort and confidence with overnight call and blood transfusions were collected via a pre-survey. A post-survey was administered to assess the simulation’s impact.

Results

The red trauma simulation led to a significant increase in residents’ confidence and comfort regarding overnight call and management of MTP. Before the simulation, many residents reported low confidence and comfort, but after the simulation, all participants reported feeling more confident and comfortable being the CA-1 anesthesia resident on overnight call and in initiating an MTP.

Conclusion

This study highlights the substantial benefits of simulation-based education in enhancing residents’ confidence and comfort regarding overnight call and an MTP.

## Introduction

The transition from intern year to the first year of anesthesia residency is a critical period for new anesthesia residents that is marked by a steep learning curve as well as the need to rapidly develop clinical proficiency. One of the most challenging aspects of this transition is taking overnight call, where residents must manage high-stakes, emergent situations often with limited backup and/or resources. Confidence and comfort during these calls are essential for both patient safety and resident well-being. Traditional didactic learning and observation-based training methods have limitations in preparing residents for the dynamic and unpredictable nature of overnight call scenarios [1].

In recent years, simulation-based education has gained prominence as a powerful tool in medical training. Simulations, which aim to replicate real-life clinical scenarios, provide a safe environment for residents to practice and refine their skills. These simulations have been shown to enhance technical proficiency, decision-making abilities, and overall clinical performance [1-3]. The impact, however, of these simulations on psychological factors such as confidence and comfort, particularly in the context of overnight call duties, has not been extensively studied. To date, there are no similar publications addressing this specific topic.

This study focuses on the use of a red trauma simulation with the need for massive blood transfusion, designed to mimic the acute, high-pressure situations encountered by anesthesia residents during overnight call. Red trauma scenarios typically involve patients with life-threatening injuries requiring quick and decisive intervention. By exposing new anesthesia residents to these realistic and challenging scenarios, simulation aims to bridge the gap between theoretical knowledge and practical application.

The primary objective of this research is to evaluate whether participation in a red trauma simulation can improve the confidence and comfort levels of new anesthesia residents when taking overnight call. Confidence, in this context, refers to the residents’ self-assurance in their ability to manage critical situations effectively. Comfort refers to their sense of ease and reduced anxiety while performing their duties under pressure. We hypothesize that the interactive nature of the red trauma simulation will enhance both of these psychological aspects, thus better preparing residents for the demands of overnight call.

This study will employ a quantitative assessment of confidence and comfort levels before and after the simulation. By assessing the evaluations of the simulation’s impact, we aim to contribute valuable insights into the optimization of residency training programs and ultimately potentially improve patient care outcomes. The findings of this research could inform the development of targeted interventions to support new anesthesia residents, fostering a more competent and resilient workforce.

## Materials and methods

Development

We implemented the red trauma simulation as part of an anesthesia resident bootcamp designed to prepare learners for managing hypotensive trauma patients requiring massive transfusions at a single center at the University of North Carolina (UNC). The target learners were anesthesia residents entering their first year of clinical anesthesia (CA-1) who had been paired with senior anesthesia residents (CA-2 or CA-3) in the operating rooms for the previous two weeks. No specific prerequisite knowledge was required. The facilitator was an experienced anesthesiologist knowledgeable in trauma management and with extensive experience in simulation-based education. Prior to the simulation, the facilitator informed learners of the chief complaint, physical setting, past medical and surgical history, social history, vital signs, physical exam findings, baseline labs, and available imaging results of the patient described in the simulation case.

The case was pilot-tested once the previous year to identify logistical challenges, such as maintaining the time-sensitive progression of vital signs and facilitating smooth transitions between simulation phases. The pilot-tested simulation was conducted with the previous year’s CA-1 class using the same personnel, equipment, and scenario. Based on this pilot, we made minor adjustments to the case timing and flow to ensure optimal fidelity.

The simulation case was designed to improve the learners’ ability to manage hypotension and massive transfusion protocols (MTPs) in a high-pressure trauma environment. We measured its success using quantitative measures and a course evaluation (Appendix D). This study was reviewed and approved by the Institutional Review Board (IRB) (approval no. 24‑1006).

Personnel

In addition to the facilitator and learners, the two other personnel needed to successfully conduct this case included a surgeon and a circulating nurse. The facilitator and the two additional personnel administering the simulation were faculty anesthesiologists, all of whom had expertise in trauma management and extensive experience in simulation-based education. The facilitator who oversaw the simulation adjusted vital signs according to the case, provided necessary products and equipment, and debriefed the learners at the end. Throughout the case, the facilitator monitored resident performance and provided prompts as needed.

Implementation

On June 13, 2024, we implemented the simulation in a UNC Hospital simulated operating room, equipped with an anesthesia machine and monitoring equipment (Appendix B). The session began with the introduction of a 25-year-old male trauma patient after an all-terrain vehicle (ATV) accident with a positive focused assessment with sonography for trauma (FAST) exam and deteriorating vital signs (Appendix A). The senior resident role was temporarily removed to create a high-pressure environment, requiring the CA-1 resident to independently manage the case. The simulation progressed through stages of induction, worsening hemodynamic instability, and resuscitation using MTP. The facilitator adjusted vital signs in real-time based on resident interventions, such as placing invasive lines, administering vasopressors, and requesting blood products. Each key moment was pre-planned with detailed case branch points to guide responses (Appendix A). The total allotted time for the simulation scenario was 10 minutes. Following the resolution of the case, a 10-minute debriefing session was held to review the resident’s performance (Appendix C). A total of 13 CA-1 resident learners participated in the simulation. Each resident was allowed to complete the simulation and debriefing session individually.

Debriefing

We utilized a structured debriefing model following the red trauma simulation to ensure that learners could reflect on their performance and connect their actions with the key learning objectives (Appendix A). Learners were guided through a reflective process using a set of standardized discussion points (Appendix C). These discussion points included operating room (OR) preparation, induction, airway management with cervical spine injury, hemodynamic instability management, MTP activation, and post-op disposition. The facilitator encouraged residents to share their thoughts on the simulation and provided feedback on both their technical and non-technical skills, such as communication and decision-making. We used an evidence-based model for debriefing, incorporating step-by-step case progression, analysis, and supplemental resources.

Assessment

We assessed the effectiveness of the simulation using pre- and post-simulation evaluation surveys (Appendix D), which measured resident confidence and comfort with overnight call and blood transfusions. The surveys included questions such as residents' confidence in taking overnight call as a CA-1 resident, their comfort in being the CA-1 anesthesia resident on call overnight, their comfort in handling emergencies during their overnight call, and their comfort with transfusion of blood products in an emergency. Additionally, the survey inquired if they were aware of the MTP and knew how to activate it. A 10-point scale was used for the answer choices to four of the seven questions, with answers ranging from “not at all confident/comfortable” to “completely confident/comfortable.” Each answer choice was assigned a score: not at all confident/comfortable = 1, up to completely confident/comfortable = 10. The post-survey was sent to all 13 of the original residents immediately after completion of the simulation. Of those 13 residents, 10 responded and filled out the post-survey. The pre- and post-survey results were then compiled and analyzed to determine the bootcamp's effectiveness in improving the residents' comfort and confidence with taking overnight call and transfusing blood products in an emergency. Additionally, we developed a critical action checklist (Appendix A) to track the completion of essential tasks such as communicating the diagnosis, ordering labs, recognizing hemodynamic instability, initiating the MTP, communicating with the surgical team, and disposition. This checklist was monitored and used as a general guide by the facilitator during the simulation.

## Results

**Figure 1:**
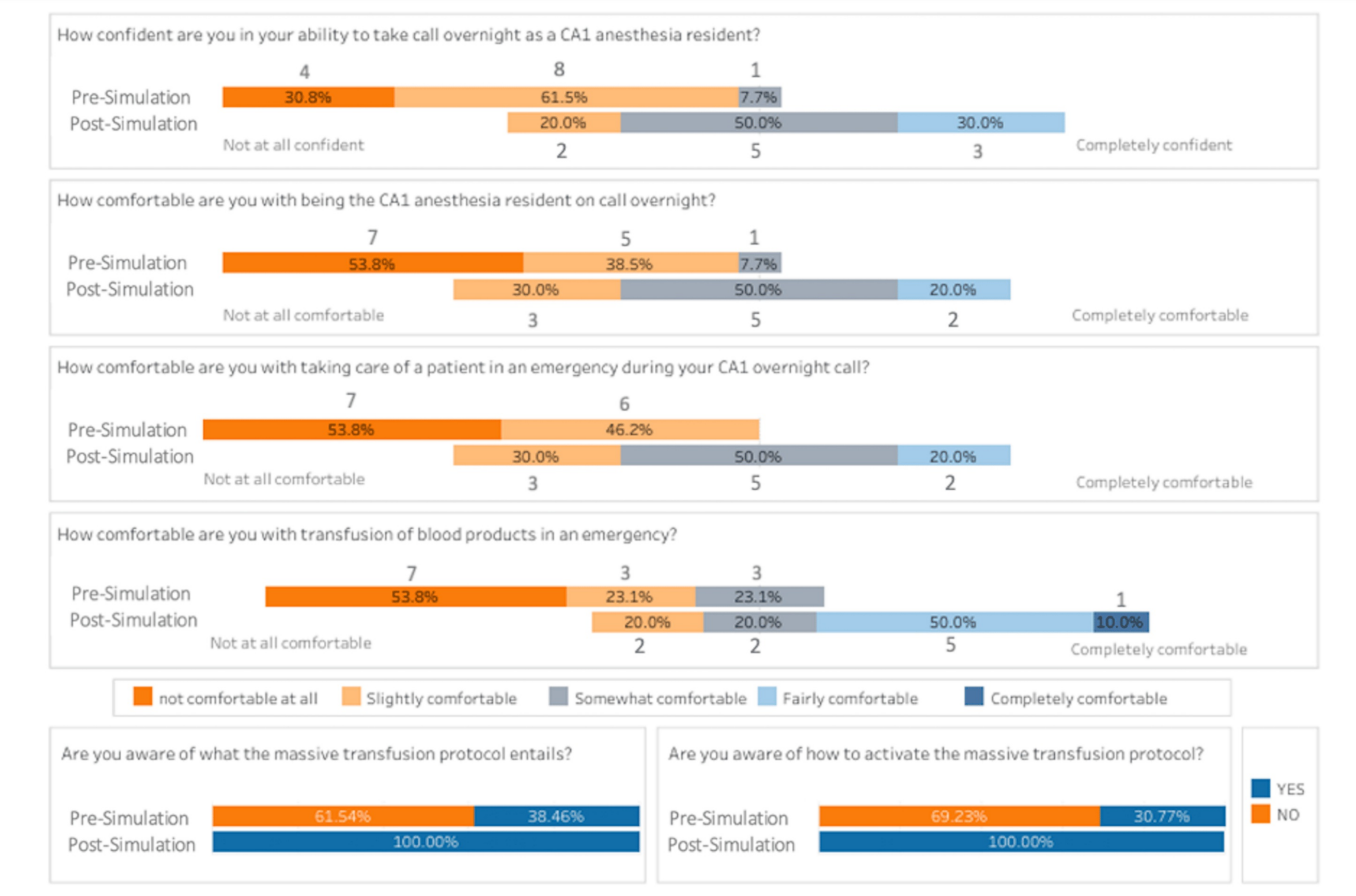
The figure shows a shift in the percentage of residents from lower to higher confidence and comfort ratings across all categories following the simulation. CA-1: first year of clinical anesthesia

**Figure 2:**
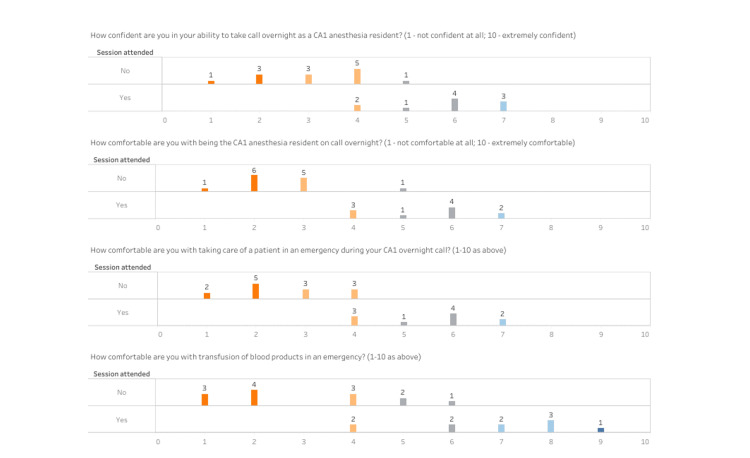
The figure illustrates the distribution of responses across different questions, with a notable increase in higher ratings post-simulation. CA-1: first year of clinical anesthesia

Pre- and post-simulation session surveys were administered to assess learners' confidence and comfort in trauma management, including their ability to initiate and manage the MTP. Before the simulation session, the residents' responses to questions about their baseline confidence and comfort in handling overnight calls, emergencies, and massive transfusions revealed a general lack in both measures. As shown in both Figures 1-2, when asked about their confidence in taking overnight calls as CA-1 residents, 30.8% of the residents indicated they were "not at all confident" (1-2 on the Likert scale), 61.5% were "slightly confident" (3-4), and only 7.7% were "somewhat confident" (5-6). Similarly, in terms of comfort with being the CA-1 resident on call overnight, 53.8% felt "not at all comfortable,” 38.5% felt “slightly comfortable,” and 7.7% felt "somewhat comfortable.” When asked about comfort in managing patients during an emergency, 53.8% of residents rated that they were “not at all comfortable,” and 46.2% reported feeling “slightly comfortable.” For comfort with blood transfusions in an emergency, 53.8% of residents felt "not at all comfortable," 23.1% felt “slightly comfortable,” and 23.1% felt "somewhat comfortable." Regarding awareness and activation of the MTP, only 38.46% of residents were aware of what the protocol entails, and 30.77% of residents were aware of how to activate the protocol.

Following the simulation session, there was a marked improvement in the residents' overall confidence and comfort. All survey participants (100%) reported feeling more confident and comfortable being the CA-1 anesthesia resident taking overnight call after attending the simulation and discussion session. Specifically, when asked about confidence in taking an overnight call, 50% of the residents now felt "somewhat confident," and 30% felt “fairly confident” (7-8). No residents rated themselves as "not at all confident,” and the percentage of those who rated “slightly confident” decreased to 20%. Regarding comfort with being the CA-1 resident on overnight call, 30% of the residents felt “slightly comfortable,” 50% recorded that they were “somewhat comfortable,” and 20% felt “fairly comfortable.” In managing patients during an emergency, 30% of the residents felt “slightly comfortable,” 50% were “somewhat comfortable,” and 20% reported being “fairly comfortable.” For comfort with blood transfusions in an emergency, 20% of residents felt "slightly comfortable," 20.0% were “somewhat comfortable,” 50% felt “fairly comfortable,” and 10.0% were "completely comfortable" (9-10). Post-simulation, 100% of the residents indicated they were fully knowledgeable of the MTP and knew how to activate it when necessary, aligning with Kirkpatrick’s Level 2 - Learning.

The results for the first four questions all showed a significant increase after the simulation session was attended versus before the simulation session. The mean difference of question one was 2.646, and this increase was statistically significant with a p-value of less than 0.001. Similarly, mean differences of questions two and three were 2.962, and these increases were both statistically significant with a p-value of less than 0.001. Finally, the mean difference of question 4 was 3.700, and this increase was statistically significant at a p-value of less than 0.001. The estimated difference before and after the simulation for awareness of MTP was 0.615, and this was statistically significant at a p-value of less than 0.001. Similarly, the estimated difference before and after the simulation for activation of MTP was 0.692, and this was statistically significant at a p-value of less than 0.001. This statistically significant improvement aligned with Kirkpatrick’s Level 1 - Reaction.

## Discussion

Overall, the red trauma simulation was successful in enhancing CA-1 residents’ confidence and comfort regarding overnight calls, emergencies, and the MTP. This educational innovation highlights the growing role of simulation-based education in bridging the gap between medical theory and practice, particularly in preparing residents for high-stakes, emergent overnight call scenarios. While traditional didactic instruction and shadowing remain foundational, these methods alone often do not equip trainees with the psychological readiness or procedural fluency required in acute care settings. In contrast, simulation-based learning creates a safe and structured environment for skill acquisition, deliberate practice, and the development of clinical judgment [4,5].

Before participating in the UNC trauma simulation bootcamp, the majority of residents reported low confidence and comfort levels with overnight calls, managing emergencies, and performing massive transfusions. Specifically, 30.8% of residents felt “not at all confident” in taking overnight calls, and 53.8% felt “not at all comfortable” being the CA-1 resident on call overnight. These findings reflect a well-documented phenomenon: the steep learning curve new residents face when transitioning to more independent clinical roles. Simulation-based interventions can effectively address this challenge. Following the red trauma simulation and debriefing session, all participants reported increased confidence and comfort. The proportion of residents who felt “somewhat confident” in taking overnight calls increased to 50%, and those who felt “fairly confident” rose to 30%. Similarly, 50% of residents reported feeling “somewhat comfortable” and 20% “fairly comfortable” as the CA-1 on call overnight.

These improvements underscore the value of high-fidelity simulation in supporting early resident development. Numerous studies have demonstrated that simulation enhances learner performance, both in technical domains (e.g., procedural skills, critical care protocols) and in the non-technical areas crucial to team-based acute care. Specifically, trauma simulations allow for safe rehearsal of rare but critical events, reinforcing rapid decision-making and interdisciplinary coordination [4,5]. The structured and immersive design of the red trauma scenario allowed residents to engage in deliberate practice, which is associated with improved retention, faster skill acquisition, and long-term performance gains [6].

In addition to technical competency, this simulation aimed to strengthen essential non-technical skills such as communication, leadership, and situational awareness. These skills are particularly critical in trauma scenarios, where the ability to manage a team under pressure can directly impact patient outcomes. Simulation training, particularly in trauma settings, has been shown in prior literature to improve non-technical skills such as communication, teamwork, and leadership - skills that are critical in managing real-life trauma situations [7].

Another critical element contributing to the effectiveness of the red trauma simulation was the structured debriefing session. The rationale behind our debriefing method stems from helping the residents understand the core lessons and necessary actions for a trauma situation, and to help residents internalize both technical and non-technical skills in trauma management. By reviewing their own thought processes, clinical decisions, and interactions with team members, learners can better understand areas of strength and opportunities for improvement. As emphasized in recent literature, structured debriefing is essential for consolidating learning and promoting self-efficacy [8]. In our implementation, the debriefing guide helped facilitate targeted discussion around key concepts such as role clarity, prioritization during massive transfusion, and communication during emergent events.

Taken together, our findings support the integration of simulation-based bootcamps into anesthesiology residency curricula, particularly during transition periods like the start of the CA-1 year. By offering early exposure to realistic clinical scenarios, these simulations help mitigate the anxiety and uncertainty commonly experienced by novice residents. Just as importantly, they foster essential clinical habits, including leadership, anticipation, and effective team coordination - skills that are not easily taught through lecture alone. As medical education increasingly shifts toward competency-based models, interventions like the red trauma simulation represent a practical and evidence-based strategy for enhancing trainee preparedness and patient safety.

Limitations

Despite the success of the simulation, several limitations were identified. The small sample size of 13 CA-1 residents limits the generalizability of the results, as the effectiveness of the intervention may vary across larger and more diverse groups. Additionally, reliance on self-reported measures of confidence and comfort introduces the possibility of bias, and without a control group, attributing the observed improvements solely to the simulation is challenging. The study also focused on short-term outcomes, with no data on the retention of skills or confidence over time, which is critical for ensuring sustained improvement in clinical performance. It has also not been extensively studied as to how an increase in comfort and/or confidence levels leads to improved patient care. The potential cost/affordability of the materials needed could limit some centers' ability to implement this simulation. Finally, since the effect of simulations on psychological factors such as confidence and comfort has not been extensively studied in the past, the surveys created by the authors had not been previously assessed for validation and/or reliability. The reliability and validity of the survey questions can be addressed with future studies.

## Conclusions

The implementation of a red trauma simulation for incoming CA-1 anesthesia residents significantly improved their confidence and comfort in managing overnight call responsibilities, particularly in high-stakes situations involving emergency transfusions. This study demonstrates that simulation-based training is a highly effective educational strategy, not only for enhancing technical skills but also for addressing the psychological readiness of early-stage residents. By replicating the intensity and complexity of real-life trauma cases in a safe, structured environment, the simulation bridged critical gaps in preparedness that traditional didactic and observational methods often fail to fill. The statistically significant improvements in the self-reported measures of confidence and comfort underscore the value of incorporating high-fidelity simulations and structured debriefing into early residency curricula. These findings support the continued integration of scenario-based simulation into anesthesiology training programs to better equip residents for the challenges of independent clinical care and to ultimately improve patient safety outcomes.

## Appendices

Appendix A: simulation description

Ideal Scenario Flow

The learner enters the room to find a patient lying on the operating room table with a cervical collar in place. They immediately place the patient on standard monitors and place an arterial line. The learner should also place at least one additional IV or central line. The learner should confirm if patient-specific blood is available or if emergency release blood is needed. Learners should ask for or confirm patient lab values (e.g., hemoglobin, arterial blood gas, metabolic profile). The learner will then induce general anesthesia with a rapid sequence induction on the patient and use a video laryngoscope to intubate the patient while minimizing neck extension by either keeping the cervical collar in place or using manual in-line stabilization of the patient’s head and neck. Learners should use pressors/fluid/blood products to treat hypotension after induction of general anesthesia. After incision, the learner should recognize hypotension likely due to uncontrolled bleeding and commence a massive transfusion protocol. The learner should give calcium for sustained hypotension after massive transfusion of blood products. Finally, the learner should make a decision to leave the patient intubated and transfer postoperatively to the ICU for higher-level care.

Initial Vital Signs

Heart rate: 110, blood pressure: 90/50, SpO_2_ 100%, temperature 35°C

Overall Setting and Appearance

Patient/mannequin lying on operating room table in cervical collar with eyes open and with standard monitors attached.

Induction and Intubation

The facilitator prompts the state to begin when the trainee begins to induce general anesthesia. Change vital signs over two minutes.

Vitals displayed: Heart rate: 120, blood pressure: 80/40, SpO_2_: 98%, end tidal CO_2_: 36, respiratory rate: 15, temperature: 34.6°C

The trainee should remove the cervical collar and provide manual inline stabilization for intubation. If the trainee places an arterial line, then display the arterial line waveform on the monitor.

Incision and Worsening Hemodynamic Stability

The facilitator prompts the state to begin when the surgeon makes an incision. Change vital signs immediately.

Vitals displayed: Heart rate: 140, blood pressure: 60/40, SPO_2_: 98%, end tidal CO_2_: 28, respiratory rate: 15, temperature: 34.1°C

Resuscitation

Facilitator prompts the state to begin when the trainee starts to treat hypotension with a vasopressor/fluid bolus. Change vital signs over two minutes.

Vitals displayed: Heart rate: 130, blood pressure: 75/50, SPO_2_: 98%, end tidal CO_2_: 36, respiratory rate: 12, temperature: 34.1°C

Massive Transfusion Protocol

Facilitators prompt the state to begin when the trainee starts to transfuse blood products. Change vital signs over two minutes.

Vitals displayed: Heart rate: 105, blood pressure: 100/60, SPO_2_: 98%, end tidal CO_2_: 34, respiratory rate: 12, temperature: 34.1°C

Resolution

The facilitator prompts the state to begin after the trainee transfuses all blood products. Change vital signs over 30 seconds.

Vitals displayed: Heart rate: 105, blood pressure: 85/55, SPO_2_: 98%, end tidal CO_2_: 36, respiratory rate: 14, temperature: 34.3°C

If the trainee asks for an arterial blood gas, give the following: pH 7.24, PaCO_2_ 36, PaO_2_ 190, HCO_3_ 18.1, Hgb 8.5, Ca 4.01. Blood pressure improves to 115/75 if the resident gives calcium.

Disposition

The facilitator prompts the state to begin after the trainee gives calcium. Change vital signs over 30 seconds.

Vitals displayed: Heart rate: 95, blood pressure: 115/75, SPO_2_: 98%, end tidal CO_2_: 34, respiratory rate: 14, temperature: 34.8°C

Discussion

Simulation is finished, and a discussion/debriefing should now take place.

Appendix B: equipment needed

Machines

Anesthesia machine, infusion pumps, rapid infuser/Belmont, defibrillator, suction machine

Airway Equipment

Endotracheal tube, bag-valve-mask, laryngoscope, laryngeal mask airway, oral airway

Monitors

Non-invasive blood pressure cuff, arterial line, capnograph, five-lead ECG, temperature probe, pulse oximeter

Medications and IV

IV start kit, normal saline, massive transfusion protocol cooler (six units PRBCs, six units FFP, one bag of platelets), labeled syringes

Others

Crash cart, Bair Hugger, mannequin

Appendix C: red trauma debriefing points

OR Preparation for Red Trauma

Call the anesthesia technician to help set up the arterial line, hotlines, and the central line. Pre-op considerations for the red trauma patient: full stomach, possible spine injuries, hypovolemia, and other injuries (pneumothorax, long bone fractures, etc.).

Induction

Induction agents: propofol, ketamine, etomidate.

RSI indications: emergency surgery, full stomach, GI pathology (gastroparesis, small bowel obstruction, GERD), increased intra-abdominal pressure (morbid obesity, ascites), pregnancy.

Cricoid pressure (Sellick maneuver): apply 10-20N of force while awake, 30-40N after asleep, to apply or not to apply is a polarized topic [9,10], probably does not cause additional neurological injury with an unstable spine [11].

Airway Management With Cervical Spine Injury

Sniffing position involves near-full extension of the atlanto-occipital and atlanto-axial joints and flexion of the lower cervical spine.

Cervical spine displacement during airway management: mask ventilation (2.93 mm), oral intubation over a lighted stylette (1.65 mm), oral intubation (1.51 mm), nasal intubation (1.20 mm) [12].

Laryngoscope insertion causes minimal c-spine movement, blade elevation causes significant extension, and intubation causes a very small amount of rotation [13]. Keeping the cervical collar on versus removing the anterior cervical collar and manual inline stabilization [14]. Glidescope is most commonly used, but also consider fiberoptic intubation.

Hemodynamic Instability Management

Differential diagnosis for hypotension: hypovolemic, vasodilatory, obstructive (tamponade, tension PTX, PE), cardiogenic (arrhythmia, ischemia), metabolic, toxicologic.

Arterial line placement. Fluid and blood administration is key. Norepinephrine has been suggested as the first-line treatment for hemorrhagic shock [15]. Inotropic agent infusion (dobutamine or epinephrine) should be used in the presence of myocardial dysfunction [15].

Massive Transfusion Protocol (MTP)

Two providers (MD or RN) are needed to check blood. Confirm patient name, MRN, date of birth, product type, blood type (if applicable), and expiration date. Product types include packed red blood cells, platelets, fresh frozen plasma, and cryoprecipitate. Do not mix blood with lactated Ringer's, dextrose-containing fluids, or hypotonic saline. All products (except platelets and cryoprecipitate) need to be warmed on a hotline. Awareness of the lethal triad of hypothermia, coagulopathy, and acidosis [16]. Manage hypothermia by increasing room temperature, using a Bair Hugger, and fluid warmers.

Post-op Disposition

Indications for ICU admission include: continued pressor support and/or massive transfusion protocol, intubated patient, implications of large volume resuscitation, and use of introducers (e.g., Cordis), which can only be managed by an ICU in many hospitals.

Appendix D: session evaluation survey questions

1. On a scale of 1-10 (1 being not confident at all, and 10 being extremely confident), how confident are you in your ability to take a call overnight as a CA1 anesthesia resident? 

2. On a scale of 1-10 (1 being not comfortable at all, and 10 being extremely comfortable), how comfortable are you with being the CA1 anesthesia resident on call overnight? 

3. On a scale of 1-10 (1 being not comfortable at all, and 10 being extremely comfortable), how comfortable are you with taking care of a patient in an emergency during your CA1 overnight call? 

4. On a scale of 1-10 (1 being not comfortable at all, and 10 being extremely comfortable), how comfortable are you with the transfusion of blood products in an emergency? 

5. Are you aware of what the massive transfusion protocol entails? 

6. Are you aware of how to activate the massive transfusion protocol? 
